# Taurine and Creatine Transporters as Potential Drug Targets in Cancer Therapy

**DOI:** 10.3390/ijms24043788

**Published:** 2023-02-14

**Authors:** Dorota Stary, Marek Bajda

**Affiliations:** 1 Department of Physicochemical Drug Analysis, Faculty of Pharmacy, Jagiellonian University Medical College, Medyczna 9 St., 30-688 Cracow, Poland; 2 Doctoral School of Medical and Health Sciences, Jagiellonian University Medical College, św. Łazarza 16 St., 31-530 Cracow, Poland

**Keywords:** taurine transporter, TauT, creatine transporter, CT-1, cancer, inhibitors

## Abstract

Cancer cells are characterized by uncontrolled growth, proliferation, and impaired apoptosis. Tumour progression could be related to poor prognosis and due to this fact, researchers have been working on novel therapeutic strategies and antineoplastic agents. It is known that altered expression and function of solute carrier proteins from the SLC6 family could be associated with severe diseases, including cancers. These proteins were noticed to play important physiological roles through transferring nutrient amino acids, osmolytes, neurotransmitters, and ions, and many of them are necessary for survival of the cells. Herein, we present the potential role of taurine (SLC6A6) and creatine (SLC6A8) transporters in cancer development as well as therapeutic potential of their inhibitors. Experimental data indicate that overexpression of analyzed proteins could be connected with colon or breast cancers, which are the most common types of cancers. The pool of known inhibitors of these transporters is limited; however, one ligand of SLC6A8 protein is currently tested in the first phase of clinical trials. Therefore, we also highlight structural aspects useful for ligand development. In this review, we discuss SLC6A6 and SLC6A8 transporters as potential biological targets for anticancer agents.

## 1. Introduction

The International Agency for Research on Cancer in 2020 published that three of the most common types of cancers were breast, lung, and colon cancers. Among them, lung and colon cancers were the most frequent causes of death in both males and females. The third reason of death was liver cancer [1]. Killing neoplasm cells without damaging normal tissues seems to be a crucial issue in antitumour therapies. The cancer therapeutic strategies could be divided into a few groups: chemotherapy, radiotherapy, immunotherapy, targeted therapy, and surgery. Therapies could also be concentrated on the specific cancer proteins or signalling pathways [2]. In the literature, it was proposed that proteins which transport some nutrients could be a promising target for cancer therapy [3,4]. It is known that transporter SLC6A14 (ATB^0,+^), which carries neutral and basic amino acids, is considered as a potential biological target for the treatment of colon, breast, and pancreas cancers [5,6,7]. In this work, we focus on the transporters for taurine (TauT, SLC6A6) and creatine (CT-1, SLC6A8) as they could play important roles in tumour growth and development [8,9]. It is known that one inhibitor of CT-1 has been investigated in clinical trials for the treatment of gastrointestinal cancer [10]. We discuss the potential of SLC6A6 and SLC6A8 transporters as the targets in therapy of cancers and analyze the features important for development of their new ligands as well.

## 2. General Overview of Taurine and Creatine Transporters

**Figure 1:**
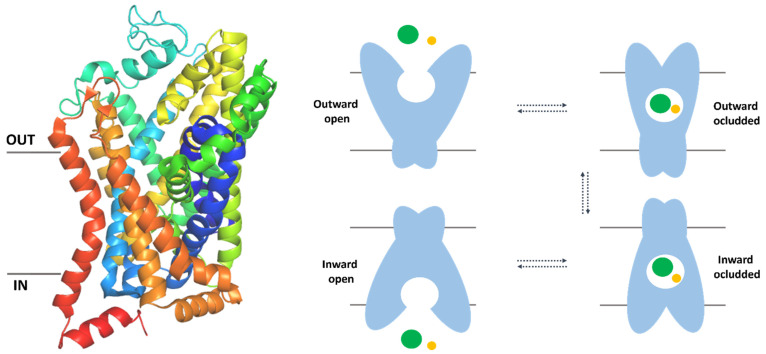
The overall structure of the SLC6 family transporters and mechanism of action. Crystal structure of dopamine transporter in outward–occluded conformation (PDB code: 4XPH). Helices are represented as cartoons (**left panel**). Schematic representation of different conformational states of transporters during transferring substrate (green sphere) and ions (yellow sphere) (**right panel**). Proteins revealed outward open state, outward occluded, inward occluded and inward open conformations [18,21].

Taurine (TauT, SLC6A6) and creatine (CT-1, SLC6A8) transporters belong to the solute carrier 6 (SLC6) family, which uses a gradient of sodium ions to actively transport substrates across the biological membranes. They constitute a part of the SLC superfamily whose members are expressed in every type of tissue. The SLC6 family involves 20 transporters (SLC6A1–SLC6A20) which are divided into four branches: GABA (γ-aminobutyric acid), monoamine, neurotransmitter amino acid, and nutrient amino acid transporters (Table 1). TauT and CT-1, together with GAT-1, GAT-2, GAT-3, and BGT-1, which are responsible for GABA turnover, are counted into the same group of GABA transporters [11,12,13]. In this branch, there is also pseudogene–SLC6A10, which is called CT-2, but its physiological functions are unclear [14]. SLC6 proteins share a general structure and consist of 12 transmembrane helices with N- and C- termini located intracellularly [15,16] (Figure 1, left panel). As mentioned, their activity is conjugated with the gradient of ion concentration. The source of energy is electrochemical potential across the biological membrane [17]. In the transport process, transporters exist in three main states. First, outward-open conformation could bind ions and substrate from the extracellular environment. Next, molecules are closed inside the binding site of the transporter with no access from both sides of the membrane. This conformation is called occluded or closed and could be divided into substates: outward occluded and inward occluded. Finally, the protein is open to the cytoplasm and releases ions and substrates [18] (Figure 1, right panel).

**Table 1:** SLC6 family transporters are divided into four groups. The table presents transporter gene names and main substrates. SLC6A10 is known as a pseudogene [11,13,19,20].

Transporter Group	Human Gene Name	Transporter Name	Main Substrates
GABA	SLC6A1	GAT-1	GABA
	SLC6A6	TauT	Taurine
	SLC6A8	CT-1	Creatine
	SLC6A10	CT-2	-
	SLC6A11	GAT-3	GABA
	SLC6A12	BGT-1	Betaine, GABA
	SLC6A13	GAT-2	GABA
Monoamines	SLC6A2	NET	Norepinephrine, dopamine
	SLC6A3	DAT	Dopamine
	SLC6A4	SERT	Serotonin
Neurotransmitter amino acids	SLC6A5	GLYT-2	Glycine
	SLC6A7	PROT	Proline
	SLC6A9	GLYT-1	Glycine
	SLC6A14	ATB^0,+^	Neutral, cationic amino acids
Nutrient amino acids	SLC6A15	B^0^AT2	Neutral amino acids
	SLC6A16	NTT5	Unknown
	SLC6A17	NTT4	Neutral amino acids
	SLC6A18	B^0^AT3	Neutral amino acids
	SLC6A19	B^0^AT1	Neutral amino acids
	SLC6A20	SIT1	Proline, pipecolate, sarcosine

The SLC6A6 gene is located on the 3p25.1 chromosome [22], whereas the human CT-1 transporter is coded by X-linked gene (Xq28) [23]. The sequence comprises 620 and 635 amino acid residues, and protein weight is equal to 69.83 and 70.52 kDa for TauT and CT-1, respectively [24,25]. The duplicated paralogue of SLC6A8 gene–SLC6A10 (CT-2), which was noticed on chromosome 16p11.2, is known as a pseudogene and its function needs further studies. Possibly, it could take part in regulation of SLC6A8 expression [20]. The main substrate of SLC6A6 protein is taurine, which participates in osmoregulation, bile acid conjugation, calcium level modulation anti-oxidation processes, and immunomodulation [26,27,28]. Taurine also plays an important role in the nervous system functioning as a GABA agonist and NMDA partial agonist through the glycine site [29,30]. For humans, taurine is a non-essential amino acid which could be synthesized from methionine or cysteine, or retrieved from dietary sources [31]. The main substrate of SLC6A8 protein is creatine, which plays a crucial role in the regulation of the cell’s energy level [32]. Creatine, similarly to taurine, is synthesized in the human body in the liver and kidney but half of the creatine organism’s demand originates from a diet [33]. The expression of the taurine transporter was confirmed in several tissues. The highest level of TauT was observed in the placenta and skeletal muscle. The intermediate expression intensity was observed in the heart, brain, lung, kidney, and pancreas, and a low amount of TauT was found in the liver [34]. Additionally, SLC6A6 transporter was noticed in leukocytes and retinal pigment epithelium [34,35]. TauT was also confirmed in the epidermis [36]. Similarly, as TauT, CT-1 was noticed in the skeletal muscle, heart, kidney, brain, colon, and prostate. SLC6A8 was also detected in the pyramidal neurons of the cerebral cortex or Purkinje cells in the cerebellar cortex [25,37]. It is worth mentioning that expression of SLC6A10 (CT-2) was noticed in the brain and testis. The exact role of this pseudogene is unclear; however, it is suggested that it may take part in autism as non-coding RNA [20].

## 3. Clinical Significance of SLC6A6 and SLC6A8 Transporters

Transporters from the SLC6 family could play multiple physiological roles. They take a part in neurotransmission, cell nutrition, and help in the maintenance of homeostasis. Impairment of their function can be associated with severe disorders [11]. In the literature, there were described diseases connected with mutations of TauT, such as Gly399Val and Ala78Glu which are connected with childhood progressive retinal degeneration and cardiomyopathy [38,39]. Studies published in 2021 showed that taurine transporter potentially could be involved in myocardial dysfunction and dilated cardiomyopathy (DCM) [40]. This analysis was connected with the identification of DCM-associated locus on chromosome 3p25.1 which encoded SLC6A6 gene, expressed among others in the heart [40]. The studies on animals showed that mice with TauT knockout displayed dilated cardiomyopathy [41]. Lack of the taurine transporter also may increase sensitivity to stress and be responsible for accelerated aging [41]. In other studies, the administration of taurine in mice with Duchenne muscular dystrophy was useful in preventing late heart dysfunction [42]. On the other hand, one of the most severe diseases connected with CT-1 transporter mutation is X-linked creatine transporter deficiency (CTD) [43], which leads to cerebral creatine deficiency syndrome (CCDS). Creatine is necessary for brain functioning, and its deficiency could be associated with intellectual disorders, behavioural changes (ADHD, autism, social anxiety), language and walking development delay, problems with coordination, and dystonia. Additionally, patients with CTD suffer from gastrointestinal problems (ulcers, vomiting, constipation) as well as cardiomyopathy, ophthalmologic problems, and bladder dysfunction [43,44]. There were noticed about 80 mutations which cause creatine transporter deficiency syndrome [43]. Interestingly, Ala404Pro substitution is connected with mild CTD, whereas Pro382Leu leads to severe symptoms [43]. In rats’ CTD model with Tyr389Cys mutation, the transporter is completely inactive [45]. Many SLC6A8 mutations cause folding defects [43]. In the case of impaired transport of creatine in the brain, the treatment strategy could be supplying creatine analogues which penetrate the blood–brain barrier, for example cyclocreatine, which is also CT-1 substrate [46]. Another disease, where a low level of CT-1 was noticed is inflammatory bowel disease (IBD) [47]. It was studied that CT-1 takes a part in wound healing and barrier formation in the intestinal epithelial cells. Patients with IBD have a lower concentration of CT-1 mRNA in the colon tissues, in comparison with the control, healthy patients. It may suggest that a lower level of CT-1 leads to the weaker barrier function of intestinal cells [47]. As it was described, severe diseases can be caused by transporters mutation and decreased function. On the other hand, overexpression and increased activity of SLC6A6 or SLC6A8 proteins were described in several types of cancer [48,49,50,51].

### 3.1. Taurine Transporter Overexpression in Cancer

Overexpression of the taurine transporter was noticed in a few types of cancers such as gastric and colorectal cancer [48,49]. The connection between taurine transporter and gastric cancer was evaluated using clinical outcomes and expression datasets, such as Gene Expression Omnibus, The Cancer Genome Atlas, and Human Protein Atlas [48]. It was found that expression of TauT was higher in gastric cancer tissue in comparison to the normal tissues. Based on immunochemistry data, it was also noticed that the intensity of SLC6A6 staining with anti-SLC6A6 antibodies was mostly stronger in cancer tissue in comparison to the normal cells [48]. High expression of TauT was found to be connected with poor prognosis and the factors such as advanced tumour stage, aggressive, especially invasive cancer. It was suggested that taurine transporter could be a diagnostic marker or potential biological target for anticancer agents [48].

Overexpression of taurine transporter was connected with colorectal cancer (CRC) progression [49]. As noticed before, this type of cancer is one of the most common neoplasms in the world [1]. It was detected that overexpression of the SLC6A6 increases survival and anti-apoptotic effects of CRC cells but does not influence their proliferation [49]. DNA microarray analysis showed that the SLC6A6 gene was highly expressed in five CRC cell lines (SW480, LoVo, DLD1, HT-29, and HCT116). This effect was not observed in healthy colonocytes [49]. Analysis of qPCR and ISH (in situ hybridization) was performed to validate the candidate colorectal cancer markers. These studies indicated TauT (SLC6A6 gene) as a novel CRC-specific surface marker [49]. Further, to develop the pathological role of the transporter in CRC, knockdown (KD) of the SLC6A6 gene in DLD1, HT-29, and HCT-15 cell lines was performed. As a result, it was observed that taurine uptake was significantly less effective in comparison to the control cell lines. The growth of tested KD cells was significantly lower, but the cell cycle showed no clear differences. Authors suggested that TauT takes a part in the regulation of the survival of CRC cells [49]. It was tested how SLC6A6 signalling could influence the side population (SP) cells and their cancer stem cell (CSC)-like properties [49]. It is known that treatment failure of CRC may be associated with the appearance of chemotherapy-resistant CSC which could present some anti-apoptotic properties [52]. In SLC6A6-KD cells, SP cells, which are characterized by higher survival rates than other cells, as well as CSC markers (LGR5, ALDHI) were diminished in comparison to the control. Additionally, the expression of colorectal cancer markers was decreased in SLC6A6-KD lines. To evaluate the prosurvival role of the TauT, Colo320DM cells were transfected with the SLC6A6 gene. The CRC cell line with high expression (Hi) of the transporter presented increased taurine uptake in comparison to the control. It was noticed that, in the late log phase, SLC6A6-Hi cells were growing, whereas control cells did not survive [49]. In numbers, about 90% of CRC Hi cells lived, when about 80% of control cells died. Additionally, in contrast to the transporter KD cells, percentage of SP cells was higher in the tested Hi lines [49]. TauT has an important role in the survival, keeping of the CSC population and their properties. These findings suggest that taurine transporter plays an important role in cancer development and could be a promising target in novel CRC therapies [49].

TauT showed higher expression in cervical cancer (CC) tissue compared to normal cervical samples [53]. Reduced miRNA level (miR-3156-3p) was identified in HPV-positive CC tissue. Downregulation of miRNA was potentially involved in cell growth promotion and lower apoptosis in HPV18-positive Hela cells, HPV16-positive SiHa, and Caski cells [53]. The bioinformatic studies proposed that the SLC6A6 gene is a possible target for miR-3156-3p. Western blot analysis showed that CC cells with over- or underexpression of this miRNA exhibited a negative correlation with SLC6A6 level. Results suggest that miR-3156-3p regulates the expression of the SLC6A6 at the post-transcriptional level. Additionally, it was found that SLC6A6 mRNA level was significantly higher in HPV-positive CC samples, in comparison to the normal cells [53]. Therefore, TauT could play a significant role in the development of cervical carcinoma. However, its exact role in this process requires future studies [53]. 

As noticed above, taurine transporter possibly makes a contribution to colon cancer development through its prosurvival and anti-apoptotic properties [49]. Overexpression of SLC6A6 was found also in gastric cancer, which was connected with poor prognosis, advanced tumour stage, and aggressiveness of cancer [48]. The role of TauT in the development and progression of tumours needs further studies, although the application of its inhibitors seems to be a novel potential therapeutic strategy, especially in colon cancer. An invention with monoclonal antibodies was also proposed, possibly conjugated with anticancer agents, which could recognize the extracellular domain of SLC6A6 [54].

### 3.2. Creatine Transporter Contribution in Cancer

The altered level and activity of creatine transporter can be connected with tumour development. Analysis of literature data showed that an elevated level of SLC6A8 was noticed in breast [55], non-small cell lung [50] or hepatocellular cancers [56]. In clinical trials, inhibition of CT-1 is tested for the treatment of colorectal carcinoma [57]. These findings indicate that the transporter is a suitable biological target for anticancer agents.

Creatine transporter was found to be overexpressed in the most aggressive subtype of breast cancer (BC)—triple-negative (TNBC), which is connected with poor prognosis [55]. TNBC is a neoplasm without the expression of receptors for oestrogens and progesterone and without amplification of human epidermal growth factor receptor 2 (HER2) [58]. It was observed that the level of CT-1 was associated with advanced tumour development, histological grade, and low condition of patients [55]. These effects possibly are caused by the anti-oxidant activity of the main substrate of CT-1. The fast-growing tumour often presents hypoxic regions which could be associated with the production of reactive oxygen species (ROS). A low level of ROS is related to cancer progression: angiogenesis and metastasis while a high level of ROS leads to cancer cell death [59]. Data analysis showed that the SLC6A8 gene was upregulated under hypoxia, which suggests that creatine can function as an antioxidant molecule in cancer cells [55]. In hypoxic TNBC cells, the activity of SLC6A8 mediated intracellular accumulation of creatine, which caused survival of cancer and decreased apoptosis by keeping homeostasis [55]. Creatine possibly takes a part in the protection of cells under hypoxic conditions by activating the AKT-ERK1/2 signalling pathway. It leads to upregulation of pro-survival Ki-67, Bcl-2, and downregulation of proapoptotic Bax protein [55]. Studies showed that the expression of the transporter under hypoxic conditions was regulated by p65/NF-κB. Tests on TNBC lines with silenced p65/NF-κB showed reduction of SLC6A8 expression. It was claimed that upregulation of SLC6A8 leads to higher creatine usage. Studies on cell models with inactive SLC6A8 transporter presented low intracellular creatine concentration [55]. To evaluate the cancer-promoting features of creatine, the cancer cell lines MDA-MB-231 with functional and nonfunctional SLC6A8 were orthotopically inoculated into mice. Animals before and during the procedure were supplemented by creatine solution injected intraperitoneally. These in vivo studies showed that mice injected with tumour cells with functional SLC6A8 presented severe disease and a higher amount of intratumour creatine as well as lower ROS levels [55]. Authors concluded that overexpression of SLC6A8 induced by hypoxic condition leads to intratumoral accumulation of creatine and helps in keeping redox homeostasis. These effects are connected with TNBC cell growing and survival [55]. Described studies showed that inhibition of CT-1 is a promising therapeutic option for the treatment of TNBC with high levels of SLC6A8 [55]. 

Interesting results were published in 2021. It was shown that therapeutic targeting of creatine transporter suppresses colon cancer progression and modulates creatine levels [57]. The studies evaluated compound RGX-202 (ompenaclid) which mimics creatine and competitively inhibits its transport by CT-1. The biological activity of RGX-202 was checked in wild-type mice and SLC6A8 knockout mice. An animal model with SLC6A8 knockout showed an undetectable level of d^3^-creatine in the heart tissue, while in WT the uptake of substrate was inhibited by RGX-202 in 75%. These findings proved ligand activity [57]. Following, it was checked how the inhibitor influenced CT-1 in tumour cells. The compound suppressed tumoral transport of labelled creatine by 50% in pancreatic tumour-bearing mice. In highly metastatic colorectal cancer cell lines (LS174T Lvm3b) under hypoxia condition, the inhibitor led to the significant reduction of the amount of phosphocreatine, which is the source of high-energy phosphate [57]. A low level of intracellular creatine or its phosphate derivative was connected with a reduced ATP level. ATP and phosphocreatine are necessary for the growth and progression of the tumour under hypoxic conditions [57]. In Lvm3b cancer cells, RGX-202 leads to the suppression of CRC growth. In animal models, the administration of the drug caused tumour growth inhibition (TGI). Lvm3b with KRAS oncogene mutation (G12D), which is a highly aggressive and metastatic cancer, were implanted subcutaneously into athymic nude mice. Oral treatment with RGX-202 began after tumours were bigger than 30 mm^3^. Inhibitors led to about 50% of TGI upon obtaining by cancer size above 500 mm^3^. Treatment improved survival of mice, from 23 to 48 days. In one among nine animals, cancer presented a complete regression response. Similar observations were noticed in the case of implanted HT29 cells: regression in case of large size of cancer (>900 mm^3^) and prolonged mice lives. Additionally, it was noticed that drug treatment increased tumour cell apoptosis. In the PDX (patient-derived xenograft) studies, the compound showed antitumour effects against several CRC subtypes [57]. 

In further studies, it was proved that CT-1 inhibition can give synergistic effects with 5-fluorouracil or leflunomide. The combination of RGX-202/5-FU led to a 99% reduction in cancer cell growth and enhanced mice survival [57]. RGX-202 was pharmaceutically optimized and under the name RGX-202-01 is currently being investigated in the first phase of clinical trials alone and in a combination with FOLFIRI with or without bevacizumab [57,60]. It was noticed that ligand increased the level of creatine in the serum and urine in patients with gastrointestinal cancer [57]. Inhibiting CT-1 and decreasing the intracellular level of creatine lead to the suppression of the cancer cell development. These findings strongly proved that the SLC6A8 transporter is a potential biological target for colorectal cancer therapy. 

During other studies, the bioinformatic analysis allowed to claim that in non-small cell lung cancer (NSCLC), the level of SLC6A8 was increased, and it was related to poor prognosis [50]. In several probes of NSCLC, high impact of the transporter was detected in comparison to the normal epithelial cells. Moreover, it was noticed that overexpression of creatine transporter promotes in vitro proliferation, migration, and invasion of NSCLC [50]. Non-small cell lung cancer lines H1299 with knockdown of SLC6A8 presented inhibited proliferation, whereas overexpression of SLC6A8 in H520 lines was connected with induced proliferation. Downregulation or upregulation of SLC6A8 activity influenced different cell cycle stages of cancer: G1 and S, respectively [50]. It was detected that H1299 cells with silenced CT-1 revealed reduced migration and invasion properties. Overexpression of SLC6A8 was correlated with high level of the invasion and migration factor—matrix metalloproteinase-9 (MMP9) [61], whereas lack of SLC6A8 gene led to downregulation of MMP9 protein [50]. Interestingly, promoting a progression could be connected with the notch signalling pathway as well as E-cadherin [50]. Therefore, blocking SLC6A8 activity in NSCLC cell lines by inhibitors seems to be an interesting field for biological studies.

The data analysis presented an abnormal SLC6A8 expression level in lung adenocarcinoma (LUAD) [51]. It was suggested that overexpression of CT-1 in LUAD is connected with poor prognosis. CT-1 possibly could be used as a cancer prognostic biomarker [51]. Creatine transporter may be a potential target in the treatment of hepatocellular cancer [56]. Study on human hepatocellular carcinoma cells (Huh-7 and Hep3B) with knockdown of the SLC6A8 gene showed that the lack of transporter leads to decrease of proliferation, induction of apoptosis, blockage of cell migration, and invasion [56]. The effect of CT-1 silencing is important in potential carcinoma therapy [56].

Literature data confirm that taurine and creatine transporter can be considered as targets in the treatment of several diseases. Diminished activity of TauT and CT-1 is mainly observed in genetic disorders [40,43,44], whereas overexpression could be associated with several types of cancers [48,49,50,51,55] (Table 2). The most important case to utilize both transporters as targets could be colorectal cancer, especially the one with poor prognosis [1]. Overexpression and over-activity of SLC6A6 transporter leads to survival of cancer cells. In the case of CT-1, inhibiting the transport of its substrate leads to tumour growth suppression [49,57]. Modifying activity of SLC6A8 by inhibitors may be tested in TNBC cells as well as NSCLC [50,55]. The exact roles of TauT and its overexpression in the development of cervical and gastric cancer need further studies (Table 2). Generally, decreasing the activity of taurine and creatine transporters could be a promising approach in antitumour therapy. As mentioned, an inhibitor of SLC6A8-RGX-202-01 is tested against gastrointestinal tract cancer, which proves the utility of such compounds [57]. There are known several groups of inhibitors, but novel compounds are expected to be developed and tested.

## 4. Inhibitors of Taurine and Creatine Transporters

The first ligands of SLC6A6 and SLC6A8 were proposed in the 20th century [62,63]. In the beginning, the impact of natural substrates and their analogues was checked [62,64]. Over the years scientists developed and tested ligands with more complex structures than simple analogues of substrates. Among them, there were compounds with aliphatic or aromatic rings, acidic groups (carboxy, sulfate, phosphate) and basic amines [65,66]. Ligands showed different biological activity in the micro or millimolar concentration range, although only one inhibitor of creatine transporter was tested against cancer (RGX-202) in clinical trials [57]. There is limited information about biological activity on carcinoma cells or animal models for other ligands, but we hope that this is a promising field to study and some of the known compounds will be useful in the development of novel, potential anticancer agents.

### 4.1. Ligands of Taurine Transporter

**Figure 2:**
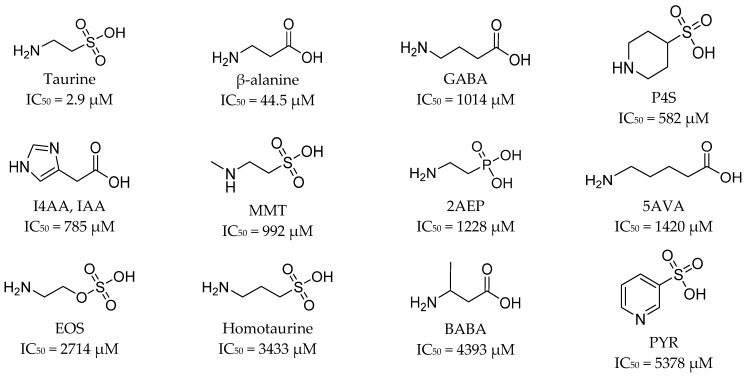
Structures of substrate analogues of taurine transporter. Their biological activity was determined by [^3^H]-taurine uptake using HEK293 cells producing TauT-GFP (green fluorescent protein) and expressed as IC_50_ values [66].

Analysis of SLC6A6 ligands, their structure, and biological activity may help in the discovery of new taurine transporter inhibitors. Taurine is β-amino acid (2-aminoethanosulfonic acid) which plays a crucial role in nervous system development [29]. TauT takes the major role in taurine transport, although transporters GAT-2 (SLC6A13) and PAT1 (SLC36A1) help in the taurine delivery system [66]. Here, we present the most important ligands of taurine transporter described in the literature. In 2019, it was investigated how GABA and taurine analogues influence [^3^H]-taurine uptake using HEK293 cells [66]. Tested ligands contained acidic moiety, carbon linker, and an amine group. As shown in Figure 2, P4S (piperidine-4-sulfonic acid), I4AA (imidazole-4-acetatic acid), and MMT (*N*-methyltaurine) showed stronger effect on transporter than GABA. It was claimed that the activity of I4AA and MTT might be connected with their cytotoxic effects [66]. The decreasing inhibition of taurine transport was observed in the following order: P4S > I4AA > MMT > 2AEP (2-aminoethylphosphonic acid) > 5AVA (5-aminovaleric acid) > EOS (2-aminoethylhydrogen sulfate) > homotaurine > BABA (β-aminobutyric acid) > PYR (3-pyridinesulfonic acid) (Figure 2). There was no influence on TauT in the case of CAHS (cis 2-aminocyclohexanecarboxylic acid), isonicotinic acid, metanilic acid, and sulfanilic acid (not shown) [66]. 

**Figure 3:**
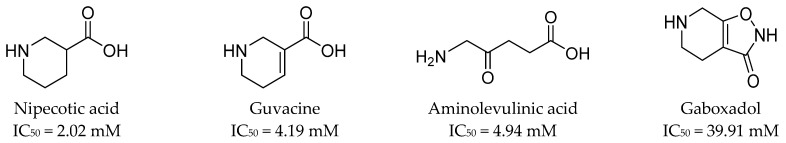
Structure of GABA and β-alanine analogues tested against TauT. Their biological activity was determined by [^3^H]-taurine uptake using SKPT cells and expressed as IC_50_ values. Compounds are sorted according to the increasing IC_50_ values [67].

In 2016, it was investigated how GABA-mimetics interacted with TauT (Figure 3) [67]. The [^3^H]-taurine uptake (Figure 3) was measured and described based on the SKPT lines. It was found that the reduction of molecule flexibility led to significantly decreased or lack of inhibitory activity. This effect was observed in case of comparing nipecotic acid (IC_50_ = 2.02 mM) and guvacine (IC_50_ = 4.19 mM) to β-alanine (IC_50_ = 0.04 mM) as well as for isonipecotic acid (uptake inhibition 2%, not significant—n.s.), gabapentin (22%, n.s.), isoguvacine (58% in 25 mM screening concentration), and gaboxadol (IC_50_ = 39.91 mM) while compared to GABA (IC_50_ = 1.07 mM). The other non-cyclic derivatives, such as aminolevulinic acid, received IC_50_ at the level of 4.94 mM [67]. 

**Figure 4:**
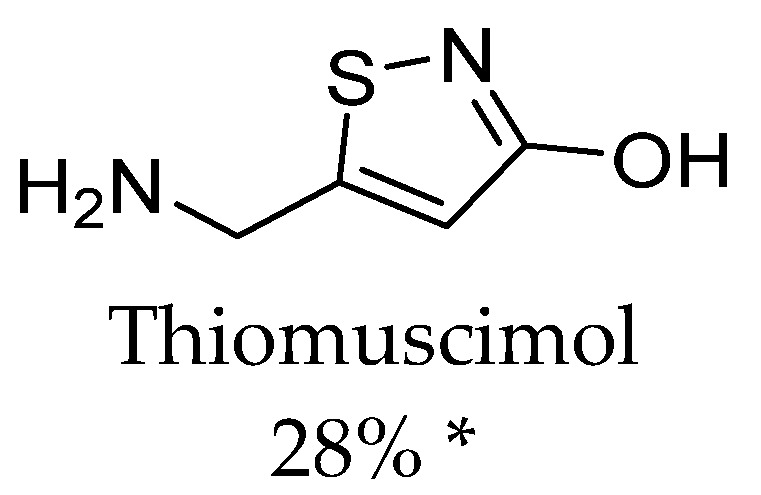
SLC6A6 ligand–thiomuscimol and impact of this GABA analogue on the taurine influx in APRE-19 cells. * Percent of inhibition of taurine uptake in 2 mM screening concentration [68].

In 2017, compounds with five-membered rings were tested against TauT [68] (Figure 4). Their ability to influence taurine uptake was checked with the ARPE-19 cells (spontaneously arising retinal epithelial cells). A significant impact on TauT was observed for I4AA (Figure 2), this effect was comparable to the one of GABA (75% and 76% taurine uptake inhibition, respectively). Low, 28% uptake inhibition was obtained for thiomuscimol (Figure 4), whereas uptake was not changed significantly in the case of THIP (gaboxadol, Figure 3) and its analogue-aza-THIP (4,5,6,7-tetrahydropyrazolo [5,4-c]pyridine-3-ol)(inhibition of 14% for both compounds) [68].

**Figure 5:**
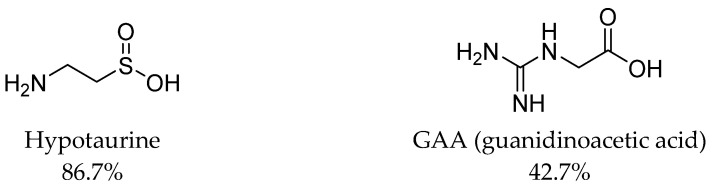
Ligands of SLC6A6 transporter. Biological activity of compounds was expressed as inhibition percentage of [^3^H]-taurine uptake using TM4 cells in 1 mM screening concentration [69].

In 2022, taurine uptake was tested by TM4–mouse Sertoli cells, which formed a blood–testis barrier [69]. Taurine transport was significantly reduced by β-alanine (95.3% uptake inhibition), hypotaurine (86.7%), GABA (76.7%), and GAA (42.7%). No significant biological activity was observed for probenecid [69]. Figure 5 presents two of the tested compounds.

To summarize, it is worth to discuss the structure of all TauT ligands. Results presented by different research groups have revealed so far that the best inhibitors for TauT are β-alanine and GABA linear analogues, especially the first ones which have two atom linkers between acidic and basic groups [66]. Active-flexible ligands were 2AEP, 5AVA, and EOS with IC_50_ values of 1228 µM, 1420 µM, and 2714 µM, respectively. More branched structure compounds, such as BABA and aminolevulinic acid, showed inhibitory activity at the level of 4.3 and 4.9 mM, respectively [66,67] (Figure 2 and Figure 3) Additionally, small compounds such as hypotaurine and GAA significantly modify taurine transport (86.7 and 42.7% uptake inhibition in 1mM concentration screening) [69] (Figure 5). Ligand with amine group in the cyclic ring presented activity as follows: P4S and nipecotic acid: IC_50_ = 528 µM and 2.02 mM, respectively (Figure 2 and Figure 3), whereas GABA analogue with cyclic ring-gabapentin was inactive similarly as cyclic alanine analogue—CAHS [66,67] (not shown). Compounds with five-membered rings in the core showed different effects. I4AA with free carboxy group has activity compared to GABA, further thiomuscimol presents 28% taurine uptake inhibition in 2 mM screening concentration, whereas muscimol was inactive (Figure 2 and Figure 4). It is worthy to notice that compound I4AA was cytotoxic [66,68]. The presence of an aromatic ring is not optimal for TauT ligands in the case of isonicotinic acid, metanilic acid, and sulfanilic acid (not shown). Additionally, PYR, which has a pyrimidine ring, presents inhibitory activity with IC_50_ above 5 mM [66] (Figure 2). It was tested that probenecid with a benzoic acid core does not significantly influence taurine transport [69]. Two-ring cores, in compounds such as THIP (gaboxadol) or aza-THIP also were unbeneficial [67,68] (Figure 3). It seems that, for new ligands, a linear structure or cyclic one with the incorporated basic group would be suitable. The possible way for the development of new taurine transporter inhibitors is to use a small five-membered ring as a core. Moreover, a basic group (primary or secondary amine or guanidine) and an acidic group (carboxyl, sulphate, or phosphate substituent) are necessary. Literature analysis showed that several ligands were tested against the SLC6A6 transporter. Possibly, some of them could have antineoplastic properties, but there is no evidence about that in the literature, and further studies are needed.

### 4.2. Ligands of the Creatine Transporter

Blocking the CT-1 activity can be used in the treatment of colorectal cancer [57]. Analysis of available inhibitors could help in new drug discovery studies. Here, we present the most important compounds tested against SLC6A8 protein.

**Figure 6:**
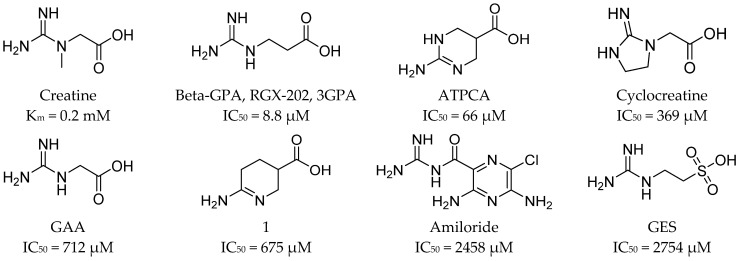
Creatine analogues tested against SLC6A8 transporter. Biological activity of compounds is expressed as IC_50_ values [70,72,73].

**Figure 7:**
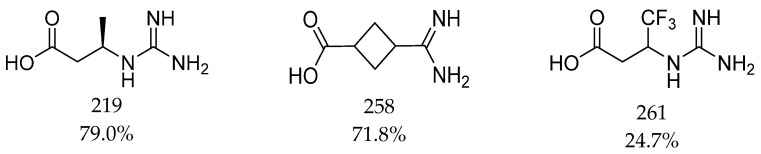
Creatine analogues tested against CT-1. Biological activity of compounds is expressed as inhibition percentage of creatine-d_3_ uptake [71].

Over the years, scientists have been interested in how different substances could modify creatine transport [62]. In 1999, a series of compounds was tested against CT-1, and it was found that uptake of creatine was inhibited by β-GPA (β-guanidinopropionic acid), cyclocreatine, γ-guanidinobutyric acid (γ-GBA), amiloride, and guanidinoethane sulfonic acid (GES). Their inhibitory activities (IC_50_) were as follows: 44.4 µM, 369.8 µM, 697.9 µM, 2458.5 µM, and 2754.1 µM, respectively (Figure 6). Creatine was characterized by obtained K_m_ of 20 µM [70]. In 2016, several compounds were proposed as inhibitors of creatine kinase or creatine transporter. It was tested how they inhibited creatine transport in heart tissue. The most effective were compounds 219 and 258, which blocked substrate transport in about 80% and 72% [71] (Figure 7). In 2018, ATPCA (2-amino-1,4,5,6-tetrahydropyrimidine-5-carboxylic acid), which is known BGT-1 substrate and inhibitor, as well as 3-guanidinopropionic acid were investigated toward the influence on the SLC6A8 activity [72]. Studies showed inhibitory potency with IC_50_ values of 66 and 8.8 µM, respectively. The observed activity is an effect of the presence of carboxy and guanidino groups separated by two or three atoms, which is characteristic for reported SLC6A8 ligands [72] (Figure 6 and Figure 7). The most important inhibitor of SLC6A8 is RGX-202 (3-GPA or β-GPA), due to its biological activity (Figure 6). RGX-202 was described as an oral small-molecule CT-1 inhibitor which is potent both in vitro and in vivo [57]. It showed multiple biological activities: it caused the reduction of phosphocreatine and ATP intracellular levels, induced tumour apoptosis, and in combination with 5-fluorouracil/leflunomide led to significant tumour regression [57]. Ligand RGX-202 was pharmaceutically optimized and as RGX-202-01 is tested against colorectal cancer in clinical trials [10].

Based on described CT-1 ligands, we found that compound β-GPA is the most effective inhibitor of this transporter (IC_50_ = 44 µM [70], or 9 µM [72]). ATPCA, which is a tetrahydropyrimidine derivative, showed less favorable IC_50_ = 66 µM [72] (Figure 6). Interestingly, ATPCA is more active than its analogue 1, which has amidine group in the place of ATPCA guanidine group [72] (Figure 6). In comparison with creatine, these ligands contain one additional carbon atom between the basic and carboxy group, which is important for ligands of CT-1. The same relationship was also observed for β-GPA and GAA (IC_50_ = 712 µM) (Figure 6) [72,73]. Similar results were noticed for compounds 219 and 258. It was found that the analogues of β-GPA with trifluoromethyl (261) (Figure 7), amine, or ethyl, substituents did not significantly inhibit creatine transport [71]. Amiloride and GES obtained IC_50_ above 2 mM (Figure 6). Describing their structure, amiloride has a pyrazine ring with amine substituents in the core with adjusted carbonylguanidine group, whereas GES contains a sulfate group isolated from a guanidine group by a two-atom linker [70]. It is interesting how this change influences the ligands’ activity because GES has the basic group isolated from acidic by a two-atom linker, as like the most active compound β-GPA, but revealed low biological activity, in the same range as more branched and devoid of acidic group amiloride [70,72] (Figure 6). Improvement of inhibitor structure and activity seems to be an interesting challenge for medicinal chemists.

## 5. Experimental Data in the Development of Novel Ligands

The first ligands of taurine and creatine transporters were discovered by classical methods in the 20th century [62,63,66,72]. Novel drugs could be proposed based on the knowledge of the structure of the biological target [74]. The exact 3D structures of TauT and CT-1 are unknown, and information about them would be helpful for the design of potential selective inhibitors and the prediction of their binding modes [24,25,75]. The overall structure of SLC6 family members could be described based on the records deposited in UniProt and Protein Data Bank (PDB) [76,77]. In the UniProt database, there was added information about amino acid sequences of all transporters from the SLC6 family from different organisms, including TauT and CT-1. Similarities between protein sequences were analyzed in the past by Beauming et al. and other groups [16,78,79,80,81,82,83]. In PDB there were deposited spatial structures of selected transporters, such as *Aquifex aeolicus* LeuT [16,84,85,86,87,88,89,90,91,92,93,94,95,96,97,98,99], *Drosphila* DAT [21,100,101,102], *human* SERT [15,103,104,105,106], GlyT-1 [107], GAT-1 [108], and B^0^AT1 [109]. Knowledge about structures of family members such as LeuT or DAT and their sequences allows for homology modelling of taurine and creatine transporters. Therefore, some possible models of CT-1 [80] or TauT [38,39] were proposed. Additionally, models of SLC6A6 and SLC6A8 were obtained by AlphaFold—the artificial intelligence approach [75]. Based on all this information, it is possible to describe potential spatial structures and important residues for transport by TauT and CT-1. These data could be helpful in novel, selective ligands development.

### 5.1. The Overall Structure of SLC6 Family Transporters

The overall structure of SLC6 family proteins, including SLC6A6 and SLC6A8, could be described as follows: they consist of 12 transmembrane helices with N- and C-termini located intracellularly [18] (Figure 1, left panel). Domains 1–10 create the core of the transporters. TM1–5 and TM6–10 were related by a pseudo-two-fold axis. The α-carbons of 130 residues from helices 1–5 could be overlaid to the atoms from helices 6–10 by rotation of 176.5° [16]. The extracellular surface of proteins is created by loops EL2, EL4, and EL6 which connect pairs of domains 3–4, 7–8, 11–12, respectively. Loop EL4 contains helical fragments and a creates V-shaped structure. Toward cytoplasm IL1 and IL5, which link helices 2 and 3, 10 and 11, respectively, are directed [15,16]. The transporter activity is conjugated with the ion gradient [17]. TauT and CT-1 for activity require two Na^+^ ions and one Cl^−^ ion per each substrate molecule [14,66]. During the uptake, the transporter adopts three main conformations: outward–open, occluded, and inward–open (Figure 1, right panel). The first place where substrates and inhibitors bind is the extracellular vestibule, called the S2 site [18]. This place is created mainly by unconserved residues. Below the S2 site, there is the extracellular gate and deeper orthosteric binding site (S1). Access to the S1 site from the cytoplasm is regulated by intracellular gate [18].

**Figure 8:**
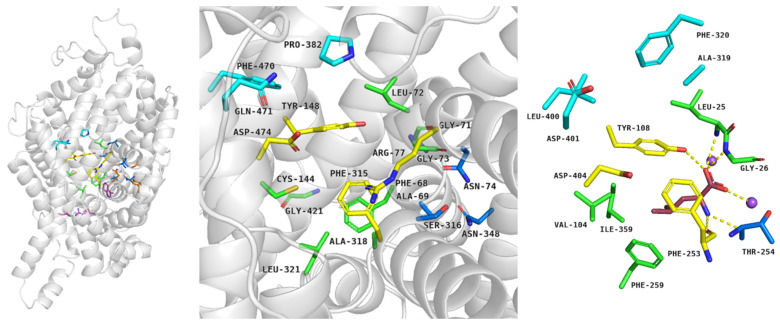
Spatial structure of transporters from SLC6 family in occluded conformation. Structure of creatine transporter was proposed by AlphaFold and analyzed in PyMOL: overall structure of CT-1 with selected residues (**left panel**), CT-1 binding site (**middle panel**). Binding mode of leucine within LeuT, PDB code: 2A65 (**right panel**). Residues coloured: blue—S2 site, yellow—extracellular gate, green—S1 site, navy blue—sodium ions binding site, pink—intracellular gate. Purple—leucine [18,75].

In detail, first, outward–open conformation is observed: the protein opens to the extracellular environment and can bind ions and substrates [18]. The residues which form pairs of the extracellular gate are distant from one another. In the TauT and CT-1 transporters, the extracellular gate is created by pairs: Arg77–Asp474 (TM1–TM10, upper part of gate) and Tyr148–Phe315 (TM3–TM6, the lower part of the gate) residues (numeration for SLC6A8) [24,25,75]. The binding sites for sodium (TM2, TM6–TM7) and chloride ions (TM1, TM6–TM8) are highly conserved among transporters [18]; we observed that only Ala69 from CT-1 is replaced by Phe58 in TauT. The S1 site is located between TM1 (Phe68, Gly71, Leu72, Gly73), TM3 (Cys144), TM6 (Ala318, Leu321), and TM8 (Gly421) (Figure 8, left and middle panel). Residues Phe68, Cys144, and Gly421 from the S1 site of creatine transporter, which are changed to Gly57, Leu134, Glu406 in case of TauT [24,25,75,78] (not shown), may have an important contribution for ligand binding. When substrates and ions are inside the transporter, residues from the extracellular gate draw together and the transporter is blocked. This conformation is called occluded or closed and could be divided into outward–occluded and inward–occluded states [18] (Figure 1, right panel). There is no information about exact taurine binding, although homology modelling studies allowed to propose binding mode of creatine. In the case of CT-1, the main substrate possibly interacts with the transporter as follows: guanidine moiety is near Tyr148, Phe315, and creates a salt bridge with deprotonated Cys144 residue. The carboxy group of creatine interacts with the main chains of Gly71 and Gly73, a hydroxy moiety of Tyr148 and sodium ion [80]. Analogical orientation in space and interactions are observed in the crystal structure of LeuT in a complex with leucine: the carboxy group interacts with a sodium ion, the main chains of Leu25, Gly26, and Tyr108, whereas the amine group is near Ala22, Phe253, Thr254. The aliphatic chain of leucine interacts with a hydrophobic pocket of LeuT, composed of Val104, Tyr108, Phe253, Phe259, and Ile359 [16] (Figure 8, right panel). In the last step, when substrates move through the transporter, interactions between residues from the intracellular gate break and substrates are released into the intracellular space [18].

It was proposed that for ligand selectivity of CT-1 transporter, two features are responsible: the presence of π–helix in TM10 and deprotonation of Cys144 [80]. It was noticed that the lack of π–helix in TM10 in SERT could determine ligand selectivity. Cys144 in the TM3 could stabilize the binding of guanidine moiety, whereas the carboxy group of creatine interacts with Gly73 and sodium ion. Between Cys144 and Na+ exists a dipole moment which leads to the proper orientation of ligands in the binding site [80]. As noticed above, residues from S1 site—Phe68, Cys144, Gly421—are replaced by Gly57, Leu134, Glu406 in TauT. Moreover, Ala69 residue in the S2 site from CT-1 is changed to Phe58 in the TauT. Substitution by small, aliphatic, or neutral residues to the amino acids with aromatic rings or acidic properties leads to differences in the binding site volume and polarity, which may be responsible for ligand selectivity. The importance of the selected residues was evaluated by mutagenesis studies [82,110,111].

### 5.2. Mutagenesis Studies on Taurine and Creatine Transporter

To discuss the role of specified residues of taurine and creatine transports, mutagenesis studies were performed [82,110,111]. Results of such experiments help in the explanation of the role of selected amino acids in the substrate selectivity and transport [82]. Additionally, biological studies and knowledge about the transporter structures may support the explanation of the roles of mutations in genetic diseases [40,43,44]. It is known that some mutations could lead to the higher or lower activity of proteins [112]. Therefore, we presented information about residue changes in TauT and CT-1 and their impact on substrate transfer.

Taurine transporter is described as an “honorary” GABA-transporter; it has a much lower affinity for GABA than GATs [82,113]. To determine the mechanism for substrate recognition mutagenesis, studies were performed. Residues such as Gly57, Phe58, Leu306, and Glu406 could take a part in the substrate transport and recognition [82]. Alignment analysis showed that these residues are conserved in TauT from human and other mammalian species. The above-mentioned four residues were different between BGT-1 and TauT. First, the mutagenesis analysis showed that substitution: G57E, F58I, L306Q, and E406C caused no detected changes in amounts of protein expression at the membrane of oocytes [82]. It was found that these four mutations lead to a decrease in [^3^H]taurine reuptake. TauT with G57E mutation presents a significant reduction in [^3^H]taurine and [^3^H]GABA uptake (reduction of 99.98% and 91.8%) in comparison to the WT transporter activity [82]. E406C mutation led to a significant increase of [^3^H]GABA uptake (3.7-fold) in comparison to the WT. This analysis suggests that Gly57 is a crucial amino acid for substrate interaction and transport. On the other hand, Glu406 would be responsible for the substrate specificity of the transporter [82]. Increased affinity for GABA in E406C mutants would be connected with the fact that transporters for GABA (BGT-1, GAT-2, and GAT-3) have cysteine in place of glutamic acid [82]. Analogically, the F58I mutant caused a higher affinity of GABA. It was suggested that Phe-to-Ile mutation reduced the bulkiness of the Phe aromatic ring, and in this way increased access of GABA to the transporter binding site [82]. The side chain of Phe58 probably could decrease the volume in the ligand binding site. Leu306 regulates substrate recognition because the L306Q mutant decreased taurine affinity and increased GABA, respectively [82]. Mutation A78E caused the decreased activity of the transporter about 95% in comparison to the WT, which was connected with retinal degeneration [39]. Molecular modelling analysis showed that this change potentially caused the rigidifying TauT structure by a salt bridge between Arg284 and Glu78 [39].

As in the case of TauT, the structure of creatine transporter was analyzed. The attention was paid to Cys144 (TM3), which seems to be crucial for CT-1 ligands selectivity. In GABA transporters, this residue is substituted by leucine [80]. To check which residues are responsible for the ligand selectivity of CT-1, mutagenesis studies were performed [110]. The uptake of GABA and creatine was tested in CT-1 with some residues from S1 replaced by the corresponding ones from GAT-1: Phe68 by Tyr (TM1), Cys144 by Leu (TM3), Ala318 by Gly (TM6), and Gly421 by Thr (TM8) [110]. It was noticed that a combination of two or three substitutions could change ligand selectivity from taurine-selective to GABA-selective properties. The lowest creatine uptake among single mutants showed Cys-to-Leu mutant. This substitution in combination with F68Y and A318G led to ~10-fold higher GABA transport than in the case of wild type [110].

In other studies, C144S/A/L mutations also showed the importance of this residue, because substitution leads to the lower uptake of [^14^C]creatine [111]. C144L was suggested to lose transporter activity in the case of creatine (15% activity of WT). In presence of GABA, the uptake of creatine by cells was reduced of 62.8% for the C144L mutant in comparison to the wild type of CT-1. It was checked that MTSEA (2-aminoethylmethanosulfonate) inhibits the activity of CT-1 at the level of 95%, which proved that cysteine is present in the CT-1 water accessible site. It was also claimed that the C144S mutant was resistant to MTSEA and revealed about 70% of the activity of WT [111]. Creatine in the presence of sodium and chloride ions prevent CT-1 inactivation by MTSEA, which could suggest that protection is connected with the transport of substrate [111].

These results showed how important experimental crystallographic and mutagenesis studies are in protein analysis. Changes in amino acid sequence help explain which residues are the most important for ligand recognition or transport [82,110,111]. Based on the information from 3D models and mutagenesis studies, it seems that for TauT activity, residues such as Gly57, Phe58, Leu134, and Glu406 are important, whereas in CT-1 the significant residues seem to be Phe68, Cys144, Gly421. This information will be helpful in the development of novel, selective ligands for both transporters [75,82,110,111]. The role of some mutations in genetic disorders needs further studies.

## 6. Conclusions

Taurine (SLC6A6) and creatine (SLC6A8) transporters belong to the GABA transporter group, from the SLC6 family. They are responsible for the transport of nutrients and osmolytes [17]. Analysis of literature data demonstrates that inhibition of these transporters could be a potential strategy for cancer treatment (Table 2). The overexpression of taurine transporter was noticed in gastric, colorectal, or cervical cancers [48,49,53]. In CRC, TauT promoted survival and prevented apoptosis of cancer cells [49]. The effect of silencing the transporter with some inhibitors should be evaluated. The exact role of SLC6A6 transporter in gastric and cervical cancer requires further studies. A high level of CT-1 was detected in triple-negative breast cancer, hepatocellular, or non-small cell lung cancers [50,55,56]. In TNBC, overexpression of creatine transporter under hypoxic conditions and accumulation of creatine led to cell growth and survival [55]. On the other hand, blocking of SLC6A8 delayed cancer progression, and one inhibitor of this transporter (RGX-202) is tested in the treatment of advanced colorectal cancer in the first phase of clinical trials [57]. However, the pool of known inhibitors of taurine or creatine transporters is limited. Compounds which bind to the TauT or CT-1 transporters mimic the substrates—taurine or creatine, respectively [62,66,67,71]. The structure of new TauT ligands could be linear or cyclic, in the latter case with an amine group incorporated into the ring. Possibly, large fragments are not beneficial in the structure of taurine transporter ligands. Similarly, in the case of the creatine transporter, the basic and acidic groups as well as the suitable conformation of the ligand is needed. The exact 3D structures of the discussed transporter are unknown, but the availability of models and mutagenesis studies could be helpful in the development of new ligands [39,75,80]. To propose novel active compounds, in silico methods could be applied. At this moment, finding potent inhibitors of SLC6A6 and SLC6A8, which may be tested in cancer cell lines, seems to be a challenge for scientists. Concluding, inhibition of taurine or creatine transporters could be a promising strategy in anticancer therapy, and therefore it is worth further detailed investigations.

## Figures and Tables

**Table 2:** Taurine and creatine transporters are associated with several types of cancer.

Gene Name	Transporter Name	Type of Cancer	Ref.
SLC6A6	Taurine transporter	Gastric cancer	[48]
		Colorectal cancer (CRC)	[49]
		Cervical cancer (CC)	[53]
SLC6A8	Creatine transporter	Triple-negative breast cancer (TNBC)	[55]
		Colorectal cancer (CRC)	[57]
		Non-small cell lung cancer (NSCLC)	[50]
		Lung adenocarcinoma (LUAD)	[51]
		Hepatocellular cancer	[56]

## Data Availability

Not applicable.

## References

[1] WHO, International Agency Research on Cancer, Cancer Today. https://gco.iarc.fr/today/data/factsheets/cancers/39-All-cancers-fact-sheet.pdf.

[2] Dembic Z., Editors A., Paveli K., Kraljevic Pavelic S., Duarte Ciceco I.F. (2020). Antitumor Drugs and Their Targets. Molecules.

[3] Enomoto K., Hotomi M. (2020). Amino Acid Transporters as Potential Therapeutic Targets in Thyroid Cancer. Endocrinol. Metab..

[4] Nałęcz K.A. (2020). Amino Acid Transporter SLC6A14 (ATB0,+)—A Target in Combined Anti-cancer Therapy. Front. Cell Dev. Biol..

[5] Mao H., Sheng J., Jia J., Wang C., Zhang S., Li H., He F. (2021). Aberrant slc6a14 expression promotes proliferation and metastasis of colorectal cancer via enhancing the jak2/stat3 pathway. OncoTargets Ther..

[6] Karunakaran S., Ramachandran S., Coothankandaswamy V., Elangovan S., Babu E., Periyasamy-Thandavan S., Gurav A., Gnanaprakasam J.P., Singh N., Schoenlein P.V. (2011). SLC6A14 (ATB 0,+) protein, a highly concentrative and broad specific amino acid transporter, is a novel and effective drug target for treatment of estrogen receptor-positive breast cancer. J. Biol. Chem..

[7] Coothankandaswamy V., Cao S., Xu Y., Prasad P.D., Singh P.K., Reynolds C.P., Yang S., Ogura J., Ganapathy V., Bhutia Y.D. (2016). Amino acid transporter SLC6A14 is a novel and effective drug target for pancreatic cancer. Br. J. Pharmacol..

[8] Zhang L., Bu P. (2022). The two sides of creatine in cancer. Trends Cell Biol..

[9] Baliou S., Kyriakopoulos A.M., Spandidos D.A., Zoumpourlis V. (2020). Role of taurine, its haloamines and its lncRNA TUG1 in both inflammation and cancer progression. On the road to therapeutics? (Review). Int. J. Oncol..

[10] A Study of RGX-202-01 as a Single Agent and as Combination Therapy in Patients with Advanced Gastrointestinal Malignancies—Full Text View—ClinicalTrials.gov, (n.d.). https://cancersearch.org/trial/NCT03597581/#.details.

[11] Pramod A.B., Foster J., Carvelli L., Henry L.K. (2013). SLC6 transporters: Structure, function, regulation, disease association and therapeutics. Mol. Aspects Med..

[12] Taslimifar M., Oparija L., Verrey F., Kurtcuoglu V., Olgac U., Makrides V. (2017). Quantifying the relative contributions of different solute carriers to aggregate substrate transport. Sci. Rep..

[13] Rudnick G., Krämer R., Blakely R.D., Murphy D.L., Verrey F. (2014). The SLC6 transporters: Perspectives on structure, functions, regulation, and models for transporter dysfunction. Pflugers Arch. Eur. J. Physiol..

[14] Saks V., Kaambre T., Guzun R., Anmann T., Sikk P., Schlattner U., Wallimann T., Aliev M., Vendelin M. (2007). Creatine and Creatine Kinase in Health and Disease. Subcell. Biochem..

[15] Coleman J.A., Green E.M., Gouaux E. (2016). X-ray structures and mechanism of the human serotonin transporter. Nature.

[16] Yamashita A., Singh S.K., Kawate T., Jin Y., Gouaux E. (2005). Crystal structure of a bacterial homologue of Na^+^/Cl^−^-dependent neurotransmitter transporters. Nature.

[17] Kristensen A.S., Andersen J., Jorgensen T.N., Sorensen L., Eriksen J., Loland C.J., Stromgaard K., Gether U. (2011). SLC6 neurotransmitter transporters: Structure, function, and regulation. Pharmacol. Rev..

[18] Łątka K., Jończyk J., Bajda M. (2020). γ-Aminobutyric acid transporters as relevant biological target: Their function, structure, inhibitors and role in the therapy of different diseases. Int. J. Biol. Macromol..

[19] Chen N.H., Reith M.E.A., Quick M.W. (2004). Synaptic uptake and beyond: The sodium-and chloride-dependent neurotransmitter transporter family SLC6. Pflugers Arch.-Eur. J. Physiol..

[20] Ndika J.D., Lusink V., Beaubrun C., Kanhai W., Martinez-Munoz C., Jakobs C., Salomons G.S. (2014). Salomons, Cloning and characterization of the promoter regions from the parent and paralogous creatine transporter genes. Gene.

[21] Wang K.H., Penmatsa A., Gouaux E. (2015). Neurotransmitter and psychostimulant recognition by the dopamine transporter. Nature.

[22] OMIM Entry—* 186854—Solute Carrier Family 6 (Neurotransmitter Transporter, Taurine), Member 6; SLC6A6, (n.d.). https://www.omim.org/entry/186854?search=slc6a6&highlight=slc6a6.

[23] OMIM Entry—* 300036—Solute Carrier Family 6 (Neurotransmitter Transporter, Creatine), Member 8; SLC6A8, (n.d.). https://www.omim.org/entry/300036?search=slc6a8&highlight=slc6a8.

[24] SLC6A6—Sodium- and Chloride-Dependent Taurine Transporter—Homo Sapiens (Human)—SLC6A6 Gene & Protein, (n.d.). https://www.uniprot.org/uniprot/P31641.

[25] SLC6A8—Sodium- and Chloride-Dependent Creatine Transporter 1—Homo Sapiens (Human)—SLC6A8 Gene & Protein, (n.d.). https://www.uniprot.org/uniprot/P48029.

[26] Marcinkiewicz J., Kontny E. (2014). Taurine and inflammatory diseases. Amino Acids..

[27] Hofmann A.F. (1999). The Continuing Importance of Bile Acids in Liver and Intestinal Disease. Arch. Intern. Med..

[28] Chen W.Q., Jin H., Nguyen M., Carr J., Lee Y.J., Hsu C.C., Faiman M.D., Schloss J.V., Wu J.Y. (2001). Role of taurine in regulation of intracellular calcium level and neuroprotective function in cultured neurons. J. Neurosci. Res..

[29] Chen C., Xia S.F., He J., Lu G., Xie Z., Han H. (2019). Roles of taurine in cognitive function of physiology, pathologies and toxication. Life Sci..

[30] Neuwirth L.S., Volpe N.P., Corwin C., Ng S., Madan N., Ferraro A.M., Furman Y., El Idrissi A. (2017). Taurine recovery of learning deficits induced by developmental Pb2+ exposure. Adv. Exp. Med. Biol..

[31] Baliou S., Kyriakopoulos A.M., Goulielmaki M., Panayiotidis M.I., Spandidos D.A., Zoumpourlis V. (2020). Significance of taurine transporter (TauT) in homeostasis and its layers of regulation (review). Mol. Med. Rep..

[32] Wyss M., Kaddurah-Daouk R. (2000). Creatine and creatinine metabolism. Physiol. Rev..

[33] Balestrino M. (2021). Role of Creatine in the Heart: Health and Disease. Nutrients.

[34] Ramamoorthy S., Leibach F.H., Mahesh V.B., Han H., Yang-Feng T., Blakely R.D., Ganapathy V. (1994). Functional characterization and chromosomal localization of a cloned taurine transporter from human placenta. Biochem. J..

[35] Schuller-Levis G.B., Park E. (2003). Taurine: New implications for an old amino acid. FEMS Microbiol. Lett..

[36] Janeke G., Siefken W., Carstensen S., Springmann G., Bleck O., Steinhart H., Höger P., Wittern K.P., Wenck H., Stäb F. (2003). Role of Taurine Accumulation in Keratinocyte Hydration. J. Investig. Dermatol..

[37] Lowe M.T.J., Faull R.L.M., Christie D.L., Waldvogel H.J. (2015). Distribution of the creatine transporter throughout the human brain reveals a spectrum of creatine transporter immunoreactivity. J. Comp. Neurol..

[38] Ansar M., Ranza E., Shetty M., Paracha S.A., Azam M., Kern I., Iwaszkiewicz J., Farooq O., Pournaras C.J., Malcles A. (2020). Taurine treatment of retinal degeneration and cardiomyopathy in a consanguineous family with SLC6A6 taurine transporter deficiency. Hum. Mol. Genet..

[39] Preising M.N., Görg B., Friedburg C., Qvartskhava N., Budde B.S., Bonus M., Toliat M.R., Pfleger C., Altmüller J., Herebian D. (2019). Biallelic mutation of human SLC6A6 encoding the taurine transporter TAUT is linked to early retinal degeneration. FASEB J..

[40] Garnier S., Harakalova M., Weiss S., Mokry M., Regitz-Zagrosek V., Hengstenberg C., Cappola T.P., Isnard R., Arbustini E., Cook S.A. (2021). Genome-wide association analysis in dilated cardiomyopathy reveals two new players in systolic heart failure on chromosomes 3p25.1 and 22q11.23. Eur. Heart J..

[41] Ito T., Oishi S., Takai M., Kimura Y., Uozumi Y., Fujio Y., Schaffer S.W., Azuma J. (2010). Cardiac and skeletal muscle abnormality in taurine transporter-knockout mice. J. Biomed. Sci..

[42] Mele A., Mantuano P., De Bellis M., Rana F., Sanarica F., Conte E., Morgese M.G., Bove M., Rolland J.F., Capogrosso R.F. (2019). A long-term treatment with taurine prevents cardiac dysfunction in mdx mice. Transl. Res..

[43] Farr C.V., El-Kasaby A., Freissmuth M., Sucic S. (2020). The Creatine Transporter Unfolded: A Knotty Premise in the Cerebral Creatine Deficiency Syndrome. Front. Synaptic Neurosci..

[44] Ghirardini E., Calugi F., Sagona G., Di Vetta F., Palma M., Battini R., Cioni G., Pizzorusso T., Baroncelli L. (2021). The Role of Preclinical Models in Creatine Transporter Deficiency: Neurobiological Mechanisms, Biomarkers and Therapeutic Development. Genes.

[45] Duran-Trio L., Fernandes-Pires G., Simicic D., Grosse J., Roux-Petronelli C., Bruce S.J., Binz P.A., Sandi C., Cudalbu C., Braissant O. (2021). A new rat model of creatine transporter deficiency reveals behavioral disorder and altered brain metabolism. Sci. Rep..

[46] Uemura T., Ito S., Masuda T., Shimbo H., Goto T., Osaka H., Wada T., Couraud P.O., Ohtsuki S. (2020). Cyclocreatine Transport by SLC6A8, the Creatine Transporter, in HEK293 Cells, a Human Blood-Brain Barrier Model Cell, and CCDSs Patient-Derived Fibroblasts. Pharm. Res..

[47] Hall C.H.T., Lee J.S., Murphy E.M., Gerich M.E., Dran R., Glover L.E., Abdulla Z.I., Skelton M.R., Colgan S.P. (2020). Creatine Transporter, Reduced in Colon Tissues From Patients With Inflammatory Bowel Diseases, Regulates Energy Balance in Intestinal Epithelial Cells, Epithelial Integrity, and Barrier Function. Gastroenterology.

[48] Wang D., Du J., Ren C., Zhou M., Xia Z. (2019). Elevated SLC6A6 expression drives tumorigenesis and affects clinical outcomes in gastric cancer. Biomark. Med..

[49] Yasunaga M., Matsumura Y. (2014). Role of SLC6A6 in promoting the survival and multidrug resistance of colorectal cancer. Sci. Rep..

[50] Feng Y., Guo X., Tang H. (2021). SLC6A8 is involved in the progression of non-small cell lung cancer through the Notch signaling pathway. Ann. Transl. Med..

[51] Fan Y., Zhou Y., Lou M., Gao Z., Li X., Yuan K. (2022). SLC6A8 is a Potential Biomarker for Poor Prognosis in Lung Adenocarcinoma. Front. Genet..

[52] Jordan C.T., Guzman M.L., Noble M. (2006). Cancer stem cells. N. Engl. J. Med..

[53] Xia Y.F., Pei G.H., Wang N., Che Y.C., Yu F.S., Yin F.F., Liu H.X., Luo B., Wang Y.K. (2017). miR-3156-3p is downregulated in HPV-positive cervical cancer and performs as a tumor-suppressive miRNA, (n.d.). Virol. J..

[54] Satofuka H., Kensuke O.H.S.E., Mukobata S., Akiyama H., Ohtsu M., Okabe Y., Murakami Y. (2019). Therapeutic Pharmaceutical Composition Employing Anti—SLC6A6 Antibody. U.S. Patent.

[55] Li Q., Liu M., Sun Y., Jin T., Zhu P., Wan X., Hou Y., Tu G. (2021). SLC6A8-mediated intracellular creatine accumulation enhances hypoxic breast cancer cell survival via ameliorating oxidative stress. J. Exp. Clin. Cancer Res..

[56] Yuan L., Wu X.J., Li W.C., Zhuo C., Xu Z., Tan C., Ma R., Wang J., Pu J. (2020). SLC6A8 Knockdown Suppresses the Invasion and Migration of Human Hepatocellular Carcinoma Huh-7 and Hep3B Cells, (n.d.). Technol. Cancer Res. Treat..

[57] Kurth I., Yamaguchi N., Andreu-Agullo C., Tian H.S., Sridhar S., Takeda S., Gonsalves F.C., Loo J.M., Barlas A., Manova-Todorova K. (2021). Therapeutic targeting of SLC6A8 creatine transporter suppresses colon cancer progression and modulates human creatine levels. Sci. Adv..

[58] Gupta G.K., Collier A.L., Lee D., Hoefer R.A., Zheleva V., van Reesema L.L.S., Tang-Tan A.M., Guye M.L., Chang D.Z., Winston J.S. (2020). Perspectives on Triple-Negative Breast Cancer: Current Treatment Strategies, Unmet Needs, and Potential Targets for Future Therapies. Cancers.

[59] Kim J., Kim J., Bae J.S. (2016). ROS homeostasis and metabolism: A critical liaison for cancer therapy. Exp. Mol. Med..

[60] A Study of RGX-202-01 as Combination Therapy in 2nd Line RAS Mutant Advanced Colorectal Cancer—Full Text View—ClinicalTrials.gov, (n.d.). https://clinicaltrials.gov/ct2/show/NCT03597581.

[61] Huang H. (2018). Matrix Metalloproteinase-9 (MMP-9) as a Cancer Biomarker and MMP-9 Biosensors: Recent Advances. Sensors.

[62] Fitch C.D., Shields R.P., Payne W.F., Dacus J.M. (1966). Creatine Metabolism in Skeletal Muscle. J. Biol. Chem..

[63] Oja S.S., Kontro P., Lähdesmäki P. (1976). Transport of taurine in the central nervous system. Adv. Exp. Med. Biol..

[64] Huxtable R.J., Laird H.E., Lippincott S.E. (1979). The transport of taurine in the heart and the rapid depletion of tissue taurine content by guanidinoethyl sulfonate. J. Pharmacol. Exp. Ther..

[65] Quesada O., Huxtable R.J., Pasantes-Morales H. (1984). Effect of guanidinoethane sulfonate on taurine uptake by rat retina. J. Neurosci. Res..

[66] Richter M., Moroniak S.J., Michel H. (2019). Identification of competitive inhibitors of the human taurine transporter TauT in a human kidney cell line. Pharm. Rep..

[67] Rasmussen R.N., Lagunas C., Plum J., Holm R., Nielsen C.U. (2016). Interaction of GABA-mimetics with the taurine transporter (TauT, Slc6a6) in hyperosmotic treated Caco-2, LLC-PK1 and rat renal SKPT cells. Eur. J. Pharm. Sci..

[68] Valembois S., Krall J., Frølund B., Steffansen B. (2017). Imidazole-4-acetic acid, a new lead structure for interaction with the taurine transporter in outer blood-retinal barrier cells. Eur. J. Pharm. Sci..

[69] Kubo Y., Ishizuka S., Ito T., Yoneyama D., Akanuma S.I., Hosoya K.I. (2022). Involvement of TauT/SLC6A6 in Taurine Transport at the Blood–Testis Barrier. Metabolites.

[70] Dai W., Vinnakota S., Qian X., Kunze D.L., Sarkar H.K. (1999). Molecular characterization of the human CRT-1 creatine transporter expressed in Xenopus oocytes. Arch. Biochem. Biophys..

[71] Martinez E.J., Tavazoie S.F. (2019). Inhibitors of Creatine Transport and Uses Thereof. U.S. Patent.

[72] Al-Khawaja A., Haugaard A.S., Marek A., Löffler R., Thiesen L., Santiveri M., Damgaard M., Bundgaard C., Frølund B., Wellendorph P. (2018). Pharmacological Characterization of [3H]ATPCA as a Substrate for Studying the Functional Role of the Betaine/GABA Transporter 1 and the Creatine Transporter. ACS Chem. Neurosci..

[73] Dodd J.R., Birch N.P., Waldvogel H.J., Christie D.L. (2010). Functional and immunocytochemical characterization of the creatine transporter in rat hippocampal neurons. J. Neurochem..

[74] Nolan T.L., Geffert L.M., Kolber B.J., Madura J.D., Surratt C.K. (2014). Discovery of Novel-scaffold monoamine transporter ligands via in silico screening with the S1 pocket of the serotonin transporter. ACS Chem. Neurosci..

[75] Varadi M., Anyango S., Deshpande M., Nair S., Natassia C., Yordanova G., Yuan D., Stroe O., Wood G., Laydon A. (2022). AlphaFold Protein Structure Database: Massively expanding the structural coverage of protein-sequence space with high-accuracy models. Nucleic Acids Res..

[76] Bateman A. (2019). UniProt: A worldwide hub of protein knowledge. Nucleic Acids Res..

[77] Berman H.M., Westbrook J., Feng Z., Gilliland G., Bhat T.N., Weissig H., Shindyalov I.N., Bourne P.E. (2000). The Protein Data Bank. Nucleic Acids Res..

[78] Beuming T., Shi L., Javitch J.A., Weinstein H. (2006). A comprehensive structure-based alignment of prokaryotic and eukaryotic neurotransmitter/Na^+^ symporters (NSS) aids in the use of the LeuT structure to probe NSS structure and function. Mol. Pharmacol..

[79] Łątka K., Jończyk J., Bajda M. (2020). Structure modeling of γ-aminobutyric acid transporters—Molecular basics of ligand selectivity. Int. J. Biol. Macromol..

[80] Colas C., Banci G., Martini R., Ecker G.F. (2020). Studies of structural determinants of substrate binding in the Creatine Transporter (CreaT, SLC6A8) using molecular models. Sci. Rep..

[81] Joseph D., Pidathala S., Mallela A.K., Penmatsa A. (2019). Structure and Gating Dynamics of Na^+^/Cl^−^ Coupled Neurotransmitter Transporters. Front. Mol. Biosci..

[82] Yahara T., Tachikawa M., Akanuma S.I., Kubo Y., Hosoya K.I. (2014). Amino acid residues involved in the substrate specificity of TauT/SLC6A6 for taurine and γ-aminobutyric acid. Biol. Pharm. Bull..

[83] Palazzolo L., Paravicini C., Laurenzi T., Adobati S., Saporiti S., Guerrini U., Gianazza E., Indiveri C., Anderson C.M.H., Thwaites D.T. (2019). SLC6A14, a Pivotal Actor on Cancer Stage: When Function Meets Structure. SLAS Discov..

[84] Kantcheva A.K., Quick M., Shi L., Winther A.M.L., Stolzenberg S., Weinstein H., Javitch J.A., Nissen P. (2013). Chloride binding site of neurotransmitter sodium symporters. Proc. Natl. Acad. Sci. USA.

[85] Kroncke B.M., Horanyi P.S., Columbus L. (2010). Structural origins of nitroxide side chain dynamics on membrane protein α-helical sites. Biochemistry.

[86] Piscitelli C.L., Gouaux E. (2012). Insights into transport mechanism from LeuT engineered to transport tryptophan. EMBO J..

[87] Wang H., Gouaux E. (2012). Substrate binds in the S1 site of the F253A mutant of LeuT, a neurotransmitter sodium symporter homologue. EMBO Rep..

[88] Singh S.K., Yamashita A., Gouaux E. (2007). Antidepressant binding site in a bacterial homologue of neurotransmitter transporters, (n.d.). Nature.

[89] Krishnamurthy H., Gouaux E. (2012). X-ray structures of LeuT in substrate-free outward-open and apo inward-open states. Nature.

[90] Wang H., Goehring A., Wang K.H., Penmatsa A., Ressler R., Gouaux E. (2013). Structural basis for action by diverse antidepressants on biogenic amine transporters. Nature.

[91] Malinauskaite L., Said S., Sahin C., Grouleff J., Shahsavar A., Bjerregaard H., Noer P., Severinsen K., Boesen T., Schiøtt B. (2016). A conserved leucine occupies the empty substrate site of LeuT in the Na^+^-free return state. Nat. Commun..

[92] Zhou Z., Zhen J., Karpowich N.K., Law C.J., Reith M.E.A., Wang D.N. (2009). Antidepressant specificity of serotonin transporter suggested by three LeuT-SSRI structures. Nat. Struct. Mol. Biol..

[93] Wang H., Elferich J., Gouaux E. (2012). Structures of LeuT in bicelles define conformation and substrate binding in a membrane-like context. Nat. Struct. Mol. Biol..

[94] Gotfryd K., Boesen T., Mortensen J.S., Khelashvili G., Quick M., Terry D.S., Missel J.W., LeVine M.V., Gourdon P., Blanchard S.C. (2020). X-ray structure of LeuT in an inward-facing occluded conformation reveals mechanism of substrate release. Nat. Commun..

[95] Quick M., Winther A.M.L., Shi L., Nissen P., Weinstein H., Javitch J.A. (2009). Binding of an octylglucoside detergent molecule in the second substrate (S2) site of LeuT establishes an inhibitor-bound conformation. Proc. Natl. Acad. Sci. USA.

[96] Campbell N.G., Shekar A., Aguilar J.I., Peng D., Navratna V., Yang D., Morley A.N., Duran A.M., Galli G., O’grady B. (2019). Structural, functional, and behavioral insights of dopamine dysfunction revealed by a deletion in SLC6A3. Proc. Natl. Acad. Sci. USA.

[97] Zhou Z., Zhen J., Karpowich N.K., Goetz R.M., Law C.J., Reith M.E.A., Wang D.N. (2007). LeuT-desipramine structure reveals how antidepressants block neurotransmitter reuptake. Science.

[98] Singh S.K., Piscitelli C.L., Yamashita A., Gouaux E. (2008). A competitive inhibitor traps LeuT in an open-to-out conformation. Science.

[99] Aguilar J.I., Cheng M.H., Font J., Schwartz A.C., Ledwitch K., Duran A., Mabry S.J., Belovich A.N., Zhu Y., Carter A.M. (2021). Psychomotor impairments and therapeutic implications revealed by a mutation associated with infantile Parkinsonism-Dystonia. Elife.

[100] Penmatsa A., Wang K.H., Gouaux E. (2013). X-ray structure of dopamine transporter elucidates antidepressant mechanism. Nature.

[101] Penmatsa A., Wang K.H., Gouaux E. (2015). X-ray structures of Drosophila dopamine transporter in complex with nisoxetine and reboxetine. Nat. Struct. Mol. Biol..

[102] Pidathala S., Mallela A.K., Joseph D., Penmatsa A. (2021). Structural basis of norepinephrine recognition and transport inhibition in neurotransmitter transporters. Nat. Commun..

[103] Plenge P., Yang D., Salomon K., Laursen L., Kalenderoglou I.E., Newman A.H., Gouaux E., Coleman J.A., Loland C.J. (2021). The antidepressant drug vilazodone is an allosteric inhibitor of the serotonin transporter. Nat. Commun..

[104] Coleman J.A., Yang D., Zhao Z., Wen P.C., Yoshioka C., Tajkhorshid E., Gouaux E. (2019). Serotonin transporter–ibogaine complexes illuminate mechanisms of inhibition and transport. Nature.

[105] Coleman J.A., Gouaux E. (2018). Structural basis for recognition of diverse antidepressants by the human serotonin transporter. Nat. Struct. Mol. Biol..

[106] Coleman J.A., Navratna V., Antermite D., Yang D., Bull J.A., Gouaux E. (2020). Chemical and structural investigation of the paroxetine-human serotonin transporter complex. Elife.

[107] Shahsavar A., Stohler P., Bourenkov G., Zimmermann I., Siegrist M., Guba W., Pinard E., Sinning S., Seeger M.A., Schneider T.R. (2021). Structural insights into the inhibition of glycine reuptake. Nature.

[108] Motiwala Z., Aduri N.G., Shaye H., Han G.W., Lam J.H., Katritch V., Cherezov V., Gati C. (2022). Structural basis of GABA reuptake inhibition. Nature.

[109] Yan R., Zhang Y., Li Y., Xia L., Guo Y., Zhou Q. (2020). Structural basis for the recognition of SARS-CoV-2 by full-length human ACE2. Science.

[110] Dodd J.R., Christie D.L. (2007). Selective Amino Acid Substitutions Convert the Creatine Transporter to a γ-Aminobutyric Acid Transporter. J. Biol. Chem..

[111] Dodd J.R., Christie D.L. (2001). Cysteine 144 in the Third Transmembrane Domain of the Creatine Transporter Is Located Close to a Substrate-binding Site. J. Biol. Chem..

[112] Backwell L., Marsh J.A. (2022). Diverse Molecular Mechanisms Underlying Pathogenic Protein Mutations: Beyond the Loss-of-Function Paradigm. Annu. Rev. Genom. Hum. Genet..

[113] Tomi M., Tajima A., Tachikawa M., Hosoya K. (2008). Function of taurine transporter (Slc6a6/TauT) as a GABA transporting protein and its relevance to GABA transport in rat retinal capillary endothelial cells. Biochim. Biophys. Acta-Biomembr..

